# Second Trimester Cervical Ectopic Pregnancy and Hemorrhage: A Case Report and Review of the Literature

**DOI:** 10.1155/2018/3860274

**Published:** 2018-08-26

**Authors:** Jessian L. Munoz, Amanda Kalan, Katherine Singh

**Affiliations:** OB/GYN and Women's Health Institute, Cleveland Clinic, 9500 Euclid Avenue, Cleveland, Ohio 44195, USA

## Abstract

Cervical ectopic pregnancies are a rare occurrence in the United States. Here we present the interdisciplinary and conservative management approach to a cervical ectopic at an advanced gestational age. In addition, we review the surgical management of hemorrhage from cervical ectopic pregnancies, which is often catastrophic and life-threatening.

## 1. Case

**Figure 1:**
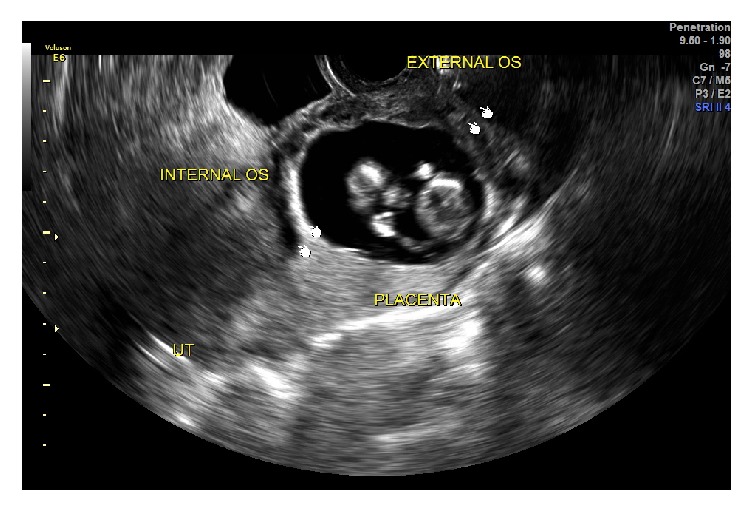
Transvaginal ultrasound: ultrasound imaging of viable cervical ectopic pregnancy. The fetus and placenta appear to be inferior or distal to the internal cervical os. The external os had about 1 cm of functional length.

**Figure 2:**
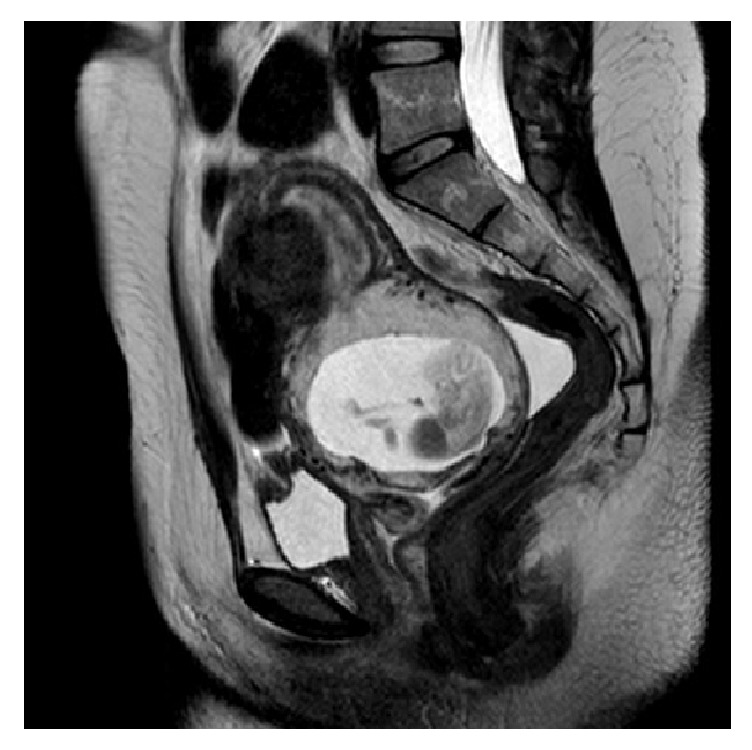
T2 weighted pelvic MRI: approximately 6.5 x 4.5 x 5 cm amniotic sac with embryo, centered in the cervix.

Index patient is a 28-year-old gravida 1 para 0 at 12 weeks and 3 days gestational age by the last menstrual period who presented for routine first trimester aneuploidy screening. During nuchal translucency ultrasound, pregnancy noted to be located within the cervix, specifically, 8mm from the external cervical os (Figure 1). Fetal heart rate was 168 at this time and there were no notable fetal anomalies. Patient denied vaginal bleeding or pain. She denied any history of gynecologic surgery. Findings were subsequently confirmed by magnetic resonance imaging (MRI) (Figure 2). The MRI showed a 6.5 x 4 x 5cm amniotic sac with embryo located within the cervix. Sterile speculum exam revealed no visible pregnancy tissue from os but enlarged and edematous cervix. HCG was 68,350 mU/mL at this time.

Given the proximity to cervical vasculature, the risk of life-threatening hemorrhage was very high. A multidisciplinary team including Reproductive Endocrinology, Maternal-Fetal Medicine, Gynecologic Oncology, and Interventional Radiology reviewed the case. At 12 weeks and 6 days gestational age, the patient was admitted to Special Delivery Unit (SDU) at the Cleveland Clinic.

Uterine artery embolization (UAE) under conscious sedation was performed with Gelfoam suspension in contrast until each uterine artery was embolized to stasis and confirmed by angiogram. Procedure was well tolerated and fetal heart rate was monitored before and after the procedure, per institutional policy. Initially the fetal heart rate was low and then not present after the procedure. Patient was then given weight-adjusted intramuscular methotrexate. On day 4 after MTX injection, patient remained asymptomatic with HCG of 19,795 mU/mL. Day 7 after MTX injection, HCG was 14,765 mU/mL. Patient was discharged on hospital day 11.

On postoperative day #14 after UAE, patient represented. Patient was febrile, with nausea/vomiting and with profuse vaginal bleeding. HCG at this time continued to show downtrend at 3360 mU/mL. Patient was taken to the operating room for dilation and curettage (D&C). Uncomplicated ultrasound-guided suction and sharp D&C were performed. Postoperatively, patient was febrile and tachycardic with evidence of pulmonary edema and acute vaginal blood loss. Patient was transferred to the intensive care unit for severe sepsis and acute kidney injury. Sepsis treated aggressively with vancomycin, clindamycin, and piperacillin-tazobactam and furosemide diuresis with clinical improvement. Antibiotics narrowed to renal-dosed piperacillin-tazobactam and then ampicillin-sulbactam. Patient was discharged in stable condition with IV antibiotic regimen. Patient was seen 2 weeks after discharge; kidney injury had resolved as well as vaginal bleeding; HCG was negative.

## 2. Discussion

Fortunately, cervical ectopic pregnancies are a rare occurrence, less than 1% of all pregnancies in the United States [1]. Unfortunately, this leads to a lack of standards of care to approach this event. Depending on the hemodynamic stability of the patient and desires for future fertility, there are different approaches both medical and surgical [2]. Irrespective of the initial management, the greatest risk of cervical ectopic pregnancies remains to be catastrophic and life-threatening hemorrhage.

Uterine artery embolization is a minimally invasive, fluoroscopy-guided procedure performed by an interventional radiologist with the goal of occluding blood flow to the uterus [3]. In 1999, Honey et al. described the first concomitant usage of UAE with intracardiac potassium chloride in the management of hemorrhage secondary to a 6-week, 3-day cervical ectopic pregnancy [4]. Marintelli et al. presented three cases of UAE in addition to D&C with preserved fertility, menses resuming in one month for two patients and one case of hysterectomy due to myoma degeneration [5].

Alternatively, the arterial branches supporting the cervix may be ligated via abdominal, laparoscopic, or vaginal approaches. Hu and colleagues described a patient who presented with profuse vaginal bleeding due to a 9-week cervical ectopic pregnancy [6]. Following exploratory laparotomy, the broad ligament was incised and the uterocervical junction was compressed with an 18F Foley catheter and tied off. The ectopic pregnancy was then excised without event. Kung et al. performed a hysteroscopic resection of 6 cervical ectopic pregnancies <9 weeks by first ligating the uterine arteries laparoscopically with a rapid absorbable suture, without the need for adjuvant therapy [7]. Alternatively, cervical blood supply may be ligated via the vaginal approach or with a high placed cervical cerclage [8, 9].

Prior to the expansion of minimally invasive methods, such as those previously described, classical methods of controlling/minimalizing blood loss from a cervical ectopic pregnancy included dilation & curettage, cervical injection of vasopressin, Foley catheter tamponade, and hysterectomy. Traditionally, hysterectomy was via the abdominal approach, yet recent data suggests laparoscopic and vaginal approaches are safe and reasonable [10, 11]. In our case, patient was nulliparous and adamantly refused hysterectomy despite the large size of the cervical pregnancy.

The case presented was unique with regard to the nature initial asymptomatic presentation, despite a gestational age almost in the second trimester. The UAE resulted in fetal demise. Later the patient presented with significant bleeding with evidence of sepsis. In order to preserve fertility, we performed a D&C, which stopped the acute bleeding episode. Yet, many different approaches exist as presented, each with advantages and disadvantages. Management should be tailored to the surgical expertise of the surgeon, patient's desires, and clinical scenario.
